# Synthesis, Optical Properties, and Sensing Applications of LaF_3_:Yb^3+^/Er^3+^/Ho^3+^/Tm^3+^ Upconversion Nanoparticles

**DOI:** 10.3390/nano10122477

**Published:** 2020-12-10

**Authors:** Hsiu-Wen Chien, Chien-Hao Huang, Chien-Hsin Yang, Tzong-Liu Wang

**Affiliations:** 1 Department of Chemical and Materials Engineering, National Kaohsiung University of Science and Technology, Kaohsiung 807, Taiwan; 2 Department of Chemical and Materials Engineering, National University of Kaohsiung, Kaohsiung 811, Taiwan; yellow1000mm@gmail.com (C.-H.H.); yangch@nuk.edu.tw (C.-H.Y.)

**Keywords:** LaF_3_, core/shell, UCNP, hexagonal, ligand exchange

## Abstract

Herein, we successfully synthesized a series of LaF_3_:Yb^3+^/Er^3+^/Ho^3+^/Tm^3+^ upconversion nanoparticles (UCNPs) and LaF_3_:Yb^3+^_0.20_, Er^3+^_0.02_@LaF_3_:Yb^3+^_0.20_ core/shell UCNPs by modifying the amount of NaOH and the reaction time. Hexagonal LaF_3_ nanocrystals with uniform particle sizes and bright UC emissions were obtained. The crystal structures of the lanthanide-doped LaF_3_ UCNPs were investigated using wide-angle X-ray diffraction. The morphologies and particle sizes of the nanocrystals were determined using transmission electron microscopy. The photoluminescence (PL) spectra of the LaF_3_ nanocrystals could be tuned by altering the doping ratio of Er^3+^, Ho^3+^, and Tm^3+^. In addition, the PL intensities increased after coating the UCNP cores with an active shell. The fluorescence intensities of the UCNPs synthesized via a one-hour reaction with the addition of 2.5 or 5 mmol NaOH increased by up to 17 times compared with the sample prepared without the addition of NaOH. By modifying the doping ratio of Yb/Tm, UV-emissive LaF_3_ nanocrystals were obtained. After surface modification by ligand exchange, the hydrophobic LaF_3_:Yb^3+^_0.20_, Er^3+^_0.02_@LaF_3_:Yb^3+^_0.20_ core/shell UCNPs became water-dispersible. These colloid UCNPs could be utilized as a fluorescent probe for the detection of Hg^2+^ ions under 980 nm near-infrared irradiation.

## 1. Introduction 

In recent years, lanthanide-doped upconversion nanoparticles (UCNPs) have attracted significant attention due to their extensive applications in various fields, such as catalysis, electronics, and biomedicine [1,2,3,4,5,6,7]. Upconversion (UC) is a nonlinear optical process characterized by the sequential absorption of multiple photons for high-energy anti-Stokes emission. Lanthanide-doped UCNPs are dilute guest–host materials that can convert near-infrared (NIR) light into higher-energy ultraviolet, visible, or NIR luminescence [8,9]. For this type of UCNP, trivalent lanthanide ions are typically used as guest ions in appropriate dielectric host lattices with dimensions of less than 100 nm. 

Owing to the 4f inner shell configuration of trivalent lanthanide ions, lanthanide-doped UCNPs with abundant and unique energy levels can exhibit numerous distinctive characteristics, including narrow emission bandwidths, long lifetimes, high resistance to photobleaching, large anti-Stokes shifts, superior photostability, and high tissue penetration depths [1,2,3,4,5,6,7,10,11]. In addition, the intensity and wavelength of UC luminescence can be adjusted by changing the lanthanide dopants and the host matrix [1,2,6]. Due to the favorable abovementioned properties, lanthanide-doped UCNPs have immense potential for biological applications [1,3,4,7,12].

The selection of appropriate host materials is crucial to obtain UCNPs that can exhibit high-efficiency emissions by reducing the nonradiative rate. Hence, the incorporation of lanthanide ions into dielectric host materials with very low-frequency phonons has attracted substantial interest. Among the hosts investigated thus far, fluoride materials have the lowest phonon cutoff energy and typically the highest UC efficiency. Lanthanide-doped rare-earth fluoride nanoparticles exhibit unique UC luminescence properties under 980 nm NIR irradiation.

Recently, rare-earth fluorides, such as binary REF_3_ and ternary AREF_4_ (RE = rare-earth, A = alkali) compounds, have attracted considerable attention because they are excellent luminescent host matrices for optically active Ln^3+^ ions [1,2,6,13,14]. Due to the exceptionally low vibration energy arising from the high ionicity of the La–F bond, LaF_3_ is considered as a promising host matrix for both, down- and UC fluorescence [13,15,16,17,18]. In particular, LaF_3_ host materials can suppress the nonradiative loss of photon energy to achieve a high quantum efficiency of luminescence.

Recently, to produce small and well-defined LaF_3_ nanocrystals for biological applications, the controlled synthesis of LaF_3_ nanoparticles has been widely studied. LaF_3_ nanocrystals with good uniformity and shape cannot be obtained by synthetic methods, including the hydrothermal route and molten salt synthesis [18,19,20,21]. In particular, no significant improvement in the fluorescence of LaF_3_ has been achieved [18,19,20,21,22,23,24,25]. Yan et al. reported a kind of LaF_3_ nanoplates with high uniformity [26]. Although these nanoplates possessed a well-defined structure and high uniformity, the products were inactive under UC luminescence. For in vivo bioimaging applications, the luminescent nanocrystals must meet certain essential requirements, such as small particle size and high brightness at NIR excitation wavelengths [2,4,27,28,29,30]. 

However, the most effective UC hosts, such as NaYF_4_, have a large size above 20 nm; this makes them difficult to be cleaned out from the body, thus limiting their applications in many biomedical fields [27,28]. In addition, although LaF_3_ nanocrystals have long been considered as effective UC host materials, LaF_3_ nanoparticles synthesized using several methods usually exhibit a plate-shaped morphology with a large size of 20–50 nm [23,24,25,31]. Therefore, the synthesis of lanthanide-doped LaF_3_ UCNPs with a uniformly small size and desirable luminescence properties remains a challenge. 

In 2013, Bao et al. fabricated uniform LaF_3_ nanocrystals with polyhedral, nanorod, and nanoplate shapes by adjusting the amount of NaOH and/or capping ligands. By doping lanthanide ions into these LaF_3_ NPs, satisfactory UC emission results were obtained [32]. However, in this study, only two types of UCNPs, that is, single-doped and core-type UCNPs, were prepared and studied, and their crystal structures remained unsatisfactory. Consequently, extensive study by researchers is underway for the fabrication of LaF_3_ UCNPs with a hexagonal prism structure, uniform size, and bright UC luminescence.

Alternatively, because mercuric ions (Hg^2+^) are toxic to human health and the environment, it is imperative to develop an efficient and highly sensitive approach for the detection of Hg^2+^. As a result, the applications of NIR-based chemosensors in bioanalysis, such as in the detection of Hg^2+^, have recently attracted considerable attention owing to the high detection sensitivity of these chemosensors [33,34].

To the best of our knowledge, few studies have reported the synthesis of uniform hexagonal lanthanide-doped core/shell LaF_3_ UCNPs with bright UC luminescence. In this study, we successfully synthesized Yb/Er co-doped LaF_3_:Yb^3+^_0.20_, Er^3+^_0.02_@LaF_3_:Yb^3+^_0.20_ core/shell UCNPs via a thermal decomposition method by modifying the amount of NaOH and the reaction time. In addition, a series of LaF_3_:Yb^3+^/Er^3+^/Ho^3+^/Tm^3+^ UCNPs were prepared for comparison. An active core/active shell strategy was adopted to restrain surface-related deactivations and intensify the energy-transfer UC of the LaF_3_:Yb^3+^/Er^3+^@LaF_3_:Yb^3+^ core/shell UCNPs [35,36,37]. The crystal structure, particle size, and morphology of the core UCNPs and core/shell UCNPs were evaluated using X-ray diffraction (XRD) and transmission electron microscopy (TEM). The emission spectrum and photoluminescence (PL) efficiency of the core UCNPs and core/shell UCNPs were investigated using a 980 nm NIR laser. The oleate-capped LaF_3_:Yb^3+^_0.20_, Er^3+^_0.02_@LaF_3_:Yb^3+^_0.20_ core/shell UCNPs were further modified with hydrophilic polyethylene glycol (PEG), and the resulting PEG-modified core/shell nanoparticles were used as chemosensors for the detection of Hg^2+^ under NIR irradiation.

## 2. Experimental Section

### 2.1. Materials

The synthesis was carried out using standard oxygen-free procedures and commercially available reagents. La_2_O_3_ (99.99%), Yb_2_O_3_ (99.99%), Er_2_O_3_ (99.99%), Ho_2_O_3_ (99.99%), Tm_2_O_3_ (99.99%), CF_3_COOH (99.99%), oleic acid (90%), octadecene (90%), HgCl_2_ (98%), and glutathione (98%) were purchased from Alfa Aesar (Ward Hill, MA, USA) and used without any further purification. NaOH (99.99%) was purchased from Sigma-Aldrich (St. Louis, MO, USA). All other chemicals used were of reagent grade.

### 2.2. Synthesis of LaF_3_:Yb^3+^/Er^3+^/Ho^3+^/Tm^3+^ UCNPs and LaF_3_:Yb^3+^/Er^3+^@LaF_3_:Yb^3+^ Core/Shell UCNPs

Yb^3+^/Er^3+^/Ho^3+^/Tm^3+^-co-doped LaF_3_ UCNPs were synthesized by the thermal decomposition approach. By adjusting the amount of NaOH and reaction time, a series of LaF_3_:Yb^3+^/Er^3+^/Ho^3+^/Tm^3+^ UCNPs were prepared using lanthanide oxides and trifluoroacetate precursors in the presence of oleic acid as a coordinating ligand and 1-octadecene as a noncoordinating solvent. The typical synthesis procedure of LaF_3_:Yb^3+^/Er^3+^@ LaF_3_:Yb^3+^ UCNPs was as follows: 0.78 mmol La_2_O_3_, 0.20 mmol Yb_2_O_3_, and 0.02 mmol Er_2_O_3_ were dissolved in 50% trifluoroacetic acid in a three-necked flask at 90 °C. Subsequently, a certain amount of NaOH was added to the abovementioned solution, and the resulting solution was evaporated to dryness under an argon gas purge. 

Next, 15 mL of oleic acid and 15 mL of 1-octadecene were added to the resulting solution in a three-necked flask followed by heating to 120 °C under magnetic stirring for 30 min to remove water and oxygen. A light-yellow solution was obtained, which was then heated to 320 °C at a rate of approximately 20 °C/min^−1^ under argon gas protection and maintained at 320 °C under vigorous stirring for approximately 1 h. The mixture was cooled to room temperature (RT) and precipitated with ethanol. The solid was collected by centrifugation, dispersed in hexane, and reprecipitated using ethanol twice to obtain the oleate-capped LaF_3_:Yb^3+^/Er^3+^ core UCNPs. The LaF_3_:Yb^3+^ shell was developed in a similar manner except that only 0.80 mmol La_2_O_3_ and 0.20 mmol Yb_2_O_3_ were used to achieve the oleate-capped LaF_3_:Yb^3+^_0.20_, Er^3+^_0.02_@LaF_3_:Yb^3+^_0.20_ core/shell UCNPs.

### 2.3. Synthesis of PEG–Imidazole

The synthesis of PEG–imidazole was described in our previous study [38]. Briefly, a certain amount of CH_3_-PEG-COOH (MW: 5000 g/mol) was dissolved in dimethylformamide (DMF) under argon protection. Subsequently, a mixture of *N,N′*-diisopropylcarbodiimide, histamine, and *N*-hydroxysuccinimide in DMF was added to the flask containing the abovementioned solution followed by heating to 75 °C. After a day of reaction, the reaction mixture was filtered to remove the solvent and recrystallized using ethanol/ether to obtain the purified product.

### 2.4. Preparation of Hydrophilic UCNPs via Ligand Exchange with PEG–Imidazole

A LaF_3_:Yb^3+^_0.20_, Er^3+^_0.02_@LaF_3_:Yb^3+^_0.20_ core/shell UCNP solution was mixed with the PEG–imidazole solution in chloroform at RT under vigorous stirring for 30 min. Next, a mixture of ethanol/hexane (1:4, *v*/*v*) was added to the abovementioned solution to produce precipitates, which were collected by filtration and then dissolved in phosphate buffer. The PEG-capped core/shell UCNPs were filtered and obtained using a polyethersulfone membrane with 0.2 µm pore size followed by centrifugation at 10,000 rpm for 10 min.

### 2.5. Assay for the Detection of Hg^2+^

The experiment for the detection of Hg^2+^ was conducted as follows. Typically, 100 mg as-synthesized UCNPs were dissolved in 250 mL of 2-(*N*-morpholino) ethanesulfonic acid buffer (0.1 M, pH = 6.0) at RT followed by the addition of a calculated amount of HgCl_2_ to produce 0, 0.2, 0.4, 0.6, and 0.8 mg/L Hg^2+^ solutions. Under 980 nm NIR irradiation, luminescence quenching spectra were obtained for each concentration. After the addition of glutathione (GSH) to the 0.8 mg/L Hg^2+^ solution, fluorescence emission measurements were performed in a similar manner. A calculated amount of GSH was dissolved in DMF to produce 0, 0.2, 0.4, 0.6, and 0.8 mg/mL GSH solutions. After separately adding the GSH solution at each concentration to the 0.8 mg/mL Hg^2+^ solution, fluorescence emission spectra were acquired for each case.

### 2.6. Characterization

Wide-angle X-ray diffractograms (WAXD) were obtained using a Bruker D8 (Bruker Axs GmbH, Karlsruhe, Germany) ADVANCE diffractometer using CuKα radiation with a step size of 0.05° and a scanning speed of 4°/min. TEM images were acquired using a JEOL JEM-1230 transmission electron microscope (JEOL Ltd., Tokyo, Japan) PL spectra were obtained using a Hitachi F-7000 fluorescence spectrophotometer. The emission spectra of the nanocrystals were obtained under 980 nm NIR excitation using a SDL-980-LM-5000T laser diode (980 nm, 3 W/cm^2^, Shanghai Dream Lasers Technology Co., Ltd., Shanghai, China).

## 3. Results and Discussion

### 3.1. Synthesis of LaF3:Yb^3+^_0.20_, Er^3+^_0.02_ core UCNPs

As the thermal decomposition method is considered the best method for the controlled synthesis of highly monodisperse nanoparticles with well-defined nanostructures [39], the LaF_3_:Yb^3+^_0.20_, Er^3+^_0.02_ core UCNPs and LaF_3_:Yb^3+^_0.20_, Er^3+^_0.02_@LaF_3_:Yb^3+^_0.20_ core/shell UCNPs were prepared via this approach. However, our preliminary study indicated that the LaF_3_:Yb^3+^/Er^3+^ core UCNPs synthesized by the thermal decomposition approach did not exhibit good morphology, crystal structure, and luminescence properties. Therefore, we attempted to optimize several reaction parameters to improve the quality of the synthesized LaF_3_:Yb^3+^/Er^3+^ core UCNPs. A significant improvement in the quality of the synthesized UCNPs was achieved after adjusting the precursor solution. The addition of NaOH to the precursor solution yielded good results; therefore, initially, we explored the optimal reaction parameters for the synthesis of the LaF_3_:Yb^3+^/Er^3+^ core UCNPs.

Based on our previous study on the synthesis of lanthanide-doped LiYF_4_-type UCNPs, we adopted a fixed ratio of LaF_3_:Yb^3+^_0.20_, Er^3+^_0.02_ and increased the reaction temperature to 320 °C. The synthesized core UCNPs were characterized by XRD, TEM, and PL spectroscopy for comparison. Herein, five experimental conditions were selected, and the results were compared: 1 h reaction without NaOH (the control sample), 2.5 mmol NaOH and 30 min reaction, 5.0 mmol NaOH and 30 min reaction, 2.5 mmol NaOH and 1 h reaction, and 5.0 mmol NaOH and 1 h reaction. 

For the addition of NaOH, we referred to the above-mentioned article [32] and presumed that the pH value could change the exact amount of CF_3_COO^−^ ions in the solution. The increase of NaOH amount would accelerate the release of CF_3_COO^−^ ions, and the fast provision of CF_3_COO^−^ ions could accelerate the growth of LaF_3_ nanocrystals. However, we found that too much NaOH and/or a longer reaction time would induce the formation of other impurities. Therefore, in our study when the amount of NaOH was increased up to 2.5 mmol and the reaction was run for one hour, some β-NaYbF_4_ was also formed, i.e., a mixture of LaF_3_ and β-NaYbF_4_ was produced. With further increasing the amount of NaOH up to 5.0 mmol and the reaction time for one hour, more β-NaYbF_4_ impurity was yielded. This implies that too much NaOH and/or a longer reaction time produces impurities in this system. Therefore, both the suitable amount of NaOH and reaction time are necessary to fine-tune to acquire the LaF_3_ type UCNPs.

### 3.2. XRD of LaF_3_:Yb^3+^_0.20_, Er^3+^_0.02_ UCNPs

**Figure 1:**
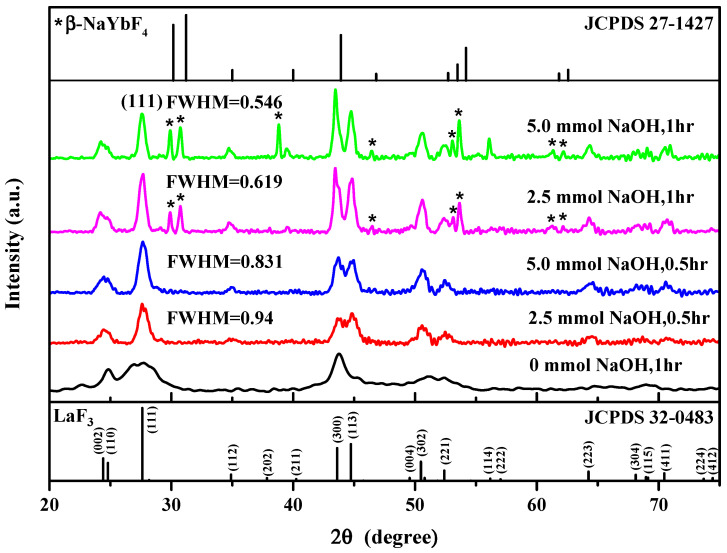
X-ray diffraction analysis of LaF_3_:Yb^3+^_0.20_, Er^3+^_0.02_ synthesized by different reaction parameters (*: β-NaYbF_4_ crystals).

To understand the effect of different experimental parameters on the crystal structure of the UCNPs, we employed XRD to determine the phase structure of the synthesized UCNPs. As shown in Figure 1, the diffraction peaks corresponding to the (002), (110), (111), (112), (211), (300), (113), (302), (221), (114), (223), (304), (115), and (411) planes of the LaF3:Yb^3+^_0.20_, Er^3+^_0.02_ UCNPs closely match the standard peaks of LaF_3_ (JCPDS No. 32-0483), confirming the successful synthesis of the LaF_3_:Yb^3+^_0.20_, Er^3+^_0.02_ UCNPs with a hexagonal crystal structure. The particle size was calculated using the Scherrer equation: Dhkl=Kλβhklcosθ, where *D_hkl_* is the mean crystallite size along the [hkl] direction, *K* is the shape factor, *λ* is the X-ray wavelength, *β_hkl_* is the line breadth (usually taken as the full width at half maximum (FWHM) of an (hkl) diffraction), and *θ* is the Bragg angle. The upper two sharp diffraction patterns indicate that the UCNPs synthesized under both the designated synthetic conditions were well crystallized; however, the pattern of the trace by-product *β*-NaYbF_4_ was also present. 

In the lowest pattern acquired for the case when the reaction was performed for 1 h without NaOH, the obtained diffraction peaks exhibited broader FWHMs, indicating a smaller crystallite size of the resulting UCNPs. After the addition of NaOH to the reaction mixture, the FWHM of the diffraction peaks became narrower, the crystallites (also referred to as grains) became larger, and the morphology of the crystal structure was improved. On the other hand, for the case where a fixed amount of NaOH was added, we observed that the longer the reaction time, the smaller the FWHM of the diffraction peak, implying the formation of bigger crystallites.

After adding NaOH and performing the reaction for 1 h, additional diffraction peaks appeared in the XRD patterns. We compared these XRD patterns with those of the possible byproducts using the information from the database and found that the additional diffraction peaks originated from the β-NaYbF_4_ crystal (JCPDS No. 27-1427). *β*-NaYbF_4_ showed a similar crystal structure (hexagonal) and UC emission spectra to those of LaF_3_-type nanoparticles, which have been reported in relevant studies [40,41,42]. Therefore, β-NaYbF_4_ would not affect the crystal morphology and peak positions of the UC emissions of the LaF_3_ UCNPs, as confirmed in the following section based on the PL spectra (Figure 4).

Based on the abovementioned results, we speculated that the addition of NaOH to the precursor solution facilitated the dissociation of CF_3_COOH and produced CF_3_COONa such that more CF_3_COO^−^ in the solution could participate in the formation reaction of LaF_3_. Hence, the growth rate of the LaF_3_ crystallites was promoted, leading to a more uniform phase structure of the LaF_3_:Yb^3+^_0.20_, Er^3+^_0.02_ UCNPs. In addition, with an increase in reaction time, more uniform and larger grains were formed. However, because of the presence of Na^+^ in the solution, some β-NaYbF_4_ was also formed.

### 3.3. Crystal Morphology of the LaF_3_:Yb^3+^_0.20_, Er^3+^_0.02_ UCNPs

**Figure 2:**
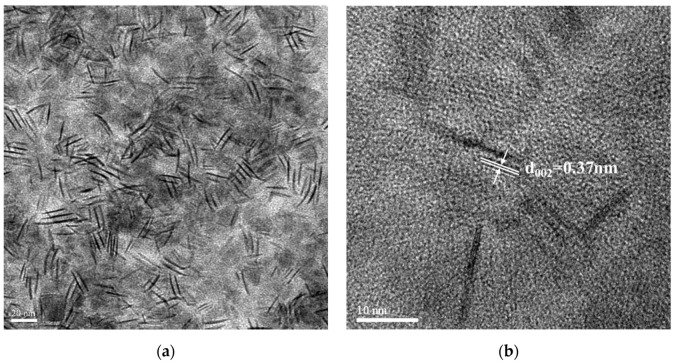

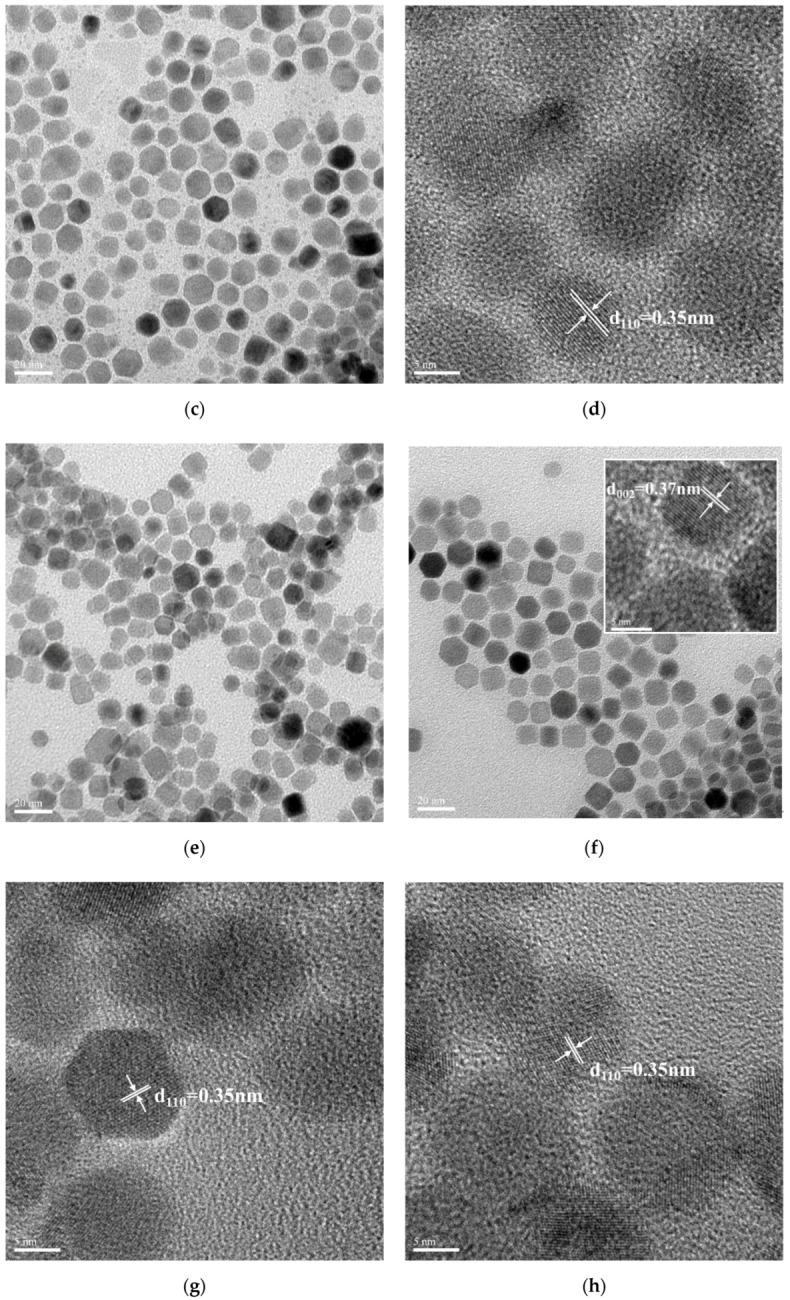

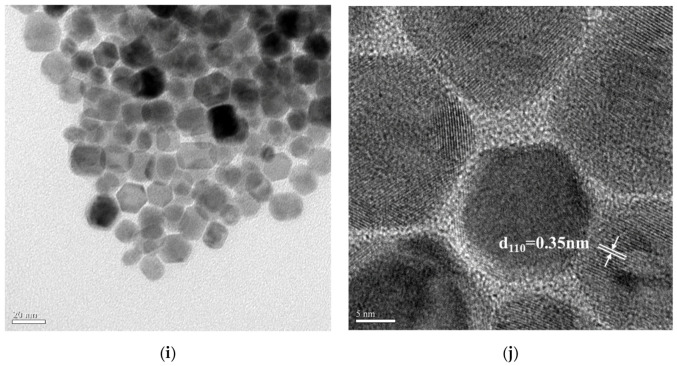
Transmission electron microscopy (TEM) bright field images and high-resolution TEM (HRTEM) images of LaF_3_:Yb^3+^_0.20_, Er^3+^_0.02_ synthesized under different reaction parameters. (**a**,**b**) 0 mmol NaOH and reaction for 1 h; (**c**,**d**) 2.5 mmol NaOH and reaction for 0.5 h; (**e**,**f**) 5.0 mmol NaOH and reaction for 0.5 h; (**g**,**h**) 2.5 mmol NaOH and reaction for 1 h; (**i**,**j**) 5.0 mmol NaOH and reaction for 1 h.

Subsequently, the effects of different reaction parameters on the crystal morphology of the UCNPs were investigated using TEM. Figure 2a,b shows the bright-field TEM image obtained with 60,000× magnification and high-resolution TEM (HRTEM) image obtained with 200,000× magnification for the LaF_3_:Yb^3+^_0.20_, Er^3+^_0.02_ UCNPs synthesized by a one-hour reaction without the addition of NaOH. Figure 2c,e,g,i presents the bright-field TEM images of the LaF_3_:Yb^3+^_0.20_, Er^3+^_0.02_ UCNPs synthesized using 2.5 mmol NaOH and a reaction time of 30 min, 2.5 mmol NaOH and a reaction time of 1 h, 5.0 mmol NaOH and a reaction time of 30 min, and 5.0 mmol NaOH and a reaction time of 1 h, respectively. The corresponding HRTEM images obtained with 300,000× magnification are shown in Figure 2d,f,h,j. All these images show that the synthesized UCNPs were single-crystal particles, and, owing to the coordination of long-chain oleic acid molecules on the crystal surface, van der Waals forces allow these UCNPs to be close-packed while being well separated from each other. As all the synthesized UCNPs are arranged in a random, isotropic manner, the TEM images (Figure 2) show a mixed type of hexagonal, rectangular, and irregular prism-like structures, confirming the formation of hexagonal-phase LaF_3_.

Figure 2a shows that the UCNPs obtained after 1 h of the thermal decomposition reaction without adding NaOH had a mixed type of structure, as mentioned above. In Figure 2a, “side-to-side” and “face-to-face” patterns were observed, indicating that the LaF_3_ crystals were arranged in a random assembly. As observed in this figure, some of the UCNPs are in the form of thin nanoplates arranged in a “face-to-face” manner; moreover, these nanoplates are hexagonal in shape when viewed from their frontal planes. This can be attributed to the fact that the synthesized UCNPs have small prism thicknesses, and, from the side view, these hexagonal prisms appear like thin plates, with a mean edge length of 16.6 ± 2.0 nm and a mean thickness of 1.4 ± 1.0 nm. 

As shown in Figure 2b, the lattice spacing was approximately 0.37 nm, which corresponds to the (002) plane of hexagonal LaF_3_ (JCPDS No. 32-0483). Subsequently, after adding 2.5 mmol NaOH followed by the thermal decomposition reaction for 30 min, more uniform hexagonal prismatic UCNPs began to appear, and the mean side length of the hexagon was approximately 7.1 ± 2.0 nm (Figure 2c). As presented in Figure 2d, the measured lattice spacing was approximately 0.35 nm, which corresponds to the (110) plane of hexagonal LaF_3_ (according to the JCPDS No. 32-0483). 

Figure 2e shows that, after adding 5.0 mmol NaOH followed by the thermal decomposition reaction for 30 min, hexagonal prismatic UCNPs with a large particle size distribution were formed. The hexagonal side length was ca. 7.5 ± 2.0 nm, and the prism height was approximately 10.5 ± 1.0 nm. Figure 2f shows the basal planes and side planes (inset) of the LaF_3_ hexagonal prism crystals. The lattice spacing of the basal planes was measured to be approximately 0.35 nm, which corresponded to the (110) plane of hexagonal LaF_3_. On the other hand, the lattice spacing of the (002) plane (inset) of hexagonal LaF_3_ was approximately 0.37 nm. 

Figure 2g shows that, after adding 2.5 mmol NaOH followed by 1 h reaction, hexagonal prismatic UCNPs with a uniform particle size were obtained. The mean side length of the hexagon was approximately 9.4 ± 1.0 nm, and the prism height was approximately 11.7 ± 1.0 nm. As shown in Figure 2h, the lattice spacing was approximately 0.35 nm, which also corresponds to the (110) plane of hexagonal LaF_3_. Figure 2i shows that after adding 5.0 mmol NaOH followed by the thermal decomposition reaction for 1 h, hexagonal prismatic UCNPs with larger sizes were achieved, indicating that the LaF_3_ crystals grew continuously under these conditions. 

**Figure 3:**
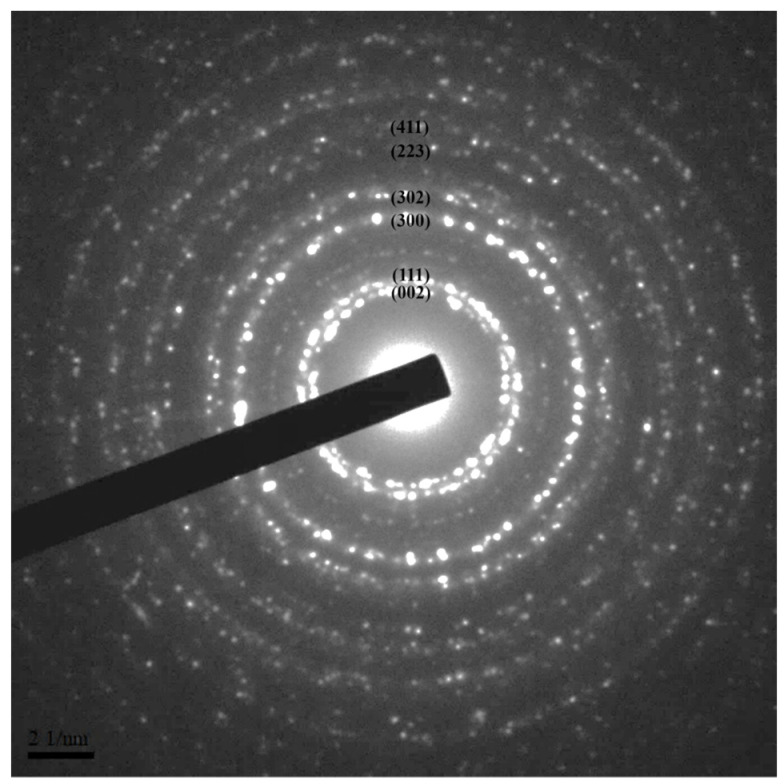
The selected-area electron diffraction (SAED) pattern of LaF_3_:Yb^3+^_0.20_, Er^3+^_0.02_ synthesized by adding 2.5 mmol NaOH and reacting for 1 h.

The side length of the hexagonal prism was approximately 10.0 ± 1.5 nm and the prism height was approximately 15.2 ± 2.0 nm. Due to the better uniformity of the LaF_3_:Yb^3+^_0.20_, Er^3+^_0.02_ core UCNPs synthesized by adding 2.5 mmol NaOH followed by reaction for 1 h, we used these UCNPs as the representative UCNPs during selected-area electron diffraction (SAED) to confirm that the synthesized UCNPs had a single-crystal structure (Figure 3); moreover, the spots of the diffraction rings were indexed to the (hkl) planes of the XRD pattern according to the standard JCPDS No. 32-0483.

From the abovementioned results, we found that the addition amount of NaOH was the key factor affecting the growth rate of the LaF_3_ grains along the (110) and (002) planes, and there was a competition between the growth of the grains along these lattice planes. The grain growth along the (110) plane can be attributed to an increase in the prism height, while the grain growth along the (002) plane indicates an increase in the side length and surface area of the basal plane of the hexagonal prism. These grain growths agree with the peak heights in the corresponding XRD patterns. 

In addition, the reaction time affected the synthesis of the LaF_3_ UCNPs, and a reaction time of 1 h resulted in the formation of more uniform and larger hexagonal crystals, as shown in Figure 2g,i. By comparing the results obtained by XRD and TEM, we speculated that the addition of NaOH would cause the dissociation of more CF_3_COOH in the precursor solution, and accordingly, primitive LaF_3_ grains were observed to grow along the (110) direction (base plane), thereby, rapidly increasing the surface area of the (002) plane. Consequently, as more CF_3_COO^−^ ions were attached to the (002) plane and participated in the reaction, the surface area of the LaF_3_ grains along the (002) plane increased, as evident from Figure 2a,c.

Hence, by comparing the lower two XRD patterns shown in Figure 1, we observed that the peak of the (002) plane emerged after adding NaOH to the reaction mixture. By comparing Figure 2c,e, we observed that, after adding up to 5.0 mmol NaOH, more uniform and larger hexagonal crystals were produced, which agrees with the FWHM values and XRD patterns shown in Figure 1. 

On the other hand, compared to the case of Figure 2c, when the amount of NaOH was kept constant and the reaction time was increased to 1 h, the growth of the crystals was promoted, resulting in crystals with better uniformity and a more defined crystal structure as shown in Figure 2g. However, when the reaction time was kept constant at 1 h and the amount of NaOH was increased up to 5.0 mmol, the growth rate of each LaF_3_ crystallite in the solution significantly varied because of the participation of more CF_3_COO^−^ ions in the reaction. As a result, compared to the case of Figure 2g, the crystals grew larger, and the particle size distribution also became larger, as shown in Figure 2i.

### 3.4. Optical Properties of the LaF_3_:Yb^3+^_0.20_, Er^3+^_0.02_ UCNPs

**Figure 4:**
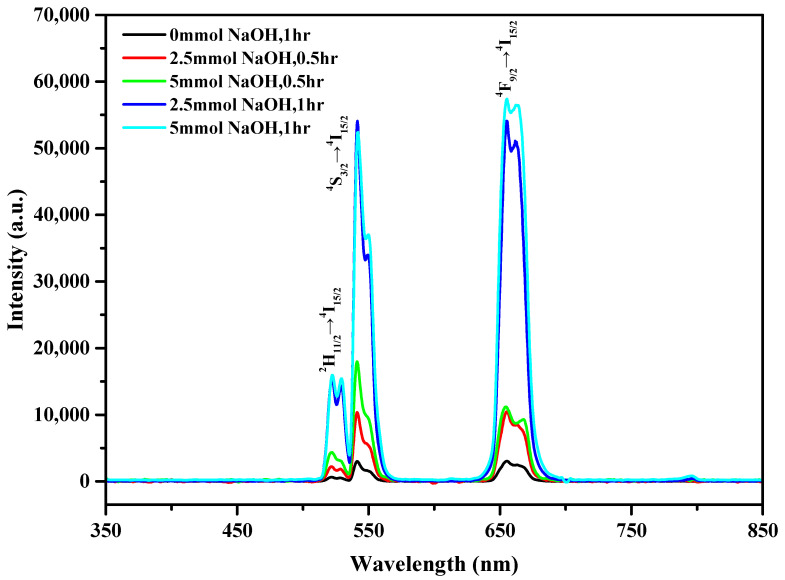
Photoluminescence spectra of LaF_3_:Yb^3+^_0.20_, Er^3+^_0.02_ core upconversion nanoparticles (UCNPs) under 980 nm excitation light.

To understand the luminescence properties of the UCNPs synthesized under different conditions, we dispersed 1 mg of UCNPs in a 2 mL mixture of n-hexane/ethanol (1:1, *v*/*v*) and analyzed the resulting solution using a fluorescence spectrometer. Figure 4 shows the PL spectra of the UCNPs obtained under the abovementioned five reaction conditions and 3 W/cm^2^ 980 nm laser irradiation. All five samples exhibited characteristic luminescence in the green and red regions. The characteristic emission peaks at 523, 542, and 656 nm were attributed to ^2^H_11/2_→^4^I_15/2_, ^4^S_3/2_→^4^I_15/2_, and ^4^F_9/2_→^4^I_15/2_ transitions, respectively, caused by the doping of Er^3+^. 

The fluorescence intensity of the control sample was the lowest among those of the five samples. The fluorescence intensities of the samples synthesized using 2.5 and 5 mmol NaOH and a reaction time of 30 min were high, approximately three to six times that of the control sample, and the fluorescence intensity of the sample prepared using 5 mmol NaOH was slightly higher than that of the sample prepared using 2.5 mmol NaOH. The fluorescence intensities of the UCNPs synthesized by the 1 h reaction with the addition of 2.5 and 5 mmol NaOH were significantly high, approximately 17 times that of the control sample. Most importantly, from Figure 4, under the abovementioned two synthetic conditions, the positions of the characteristic emission peaks did not change because β-NaYbF_4_ exhibited UC emission peaks similar to those of the LaF_3_ nanocrystals [40,41,42].

The increase in the fluorescence intensities of the UCNPs can be explained based on the XRD and TEM results. The fluorescence principle of UCNPs is based on an energy-transfer UC process, in which a sensitizer (Yb^3+^ in this case) absorbs a pump photon and is excited from the ground state to its metastable energy level. The harvested energy of the sensitizer is then transferred to the ground state and the excited state of the activator (Er^3+^ in the present case). Subsequently, the transferred energy excites the activator to its upper emitting state, and the sensitizer returns to the ground state twice [43]. 

The energy is released in the form of light. Hence, the defects in the LaF_3_ host material have a considerable influence on the fluorescence efficiency of UCNPs. As observed in Figure 1, the XRD peaks of the UCNPs formed by the addition of NaOH exhibit a narrower FWHM, which indicates that the as-prepared UCNPs are larger, and more defined grains and fewer surface defects can avoid slight energy losses during the energy transfer. As observed in Figure 2, the morphology of the UCNPs synthesized by the reaction involving the addition of NaOH changed from the original plate shape to a hexagonal prism shape. This may suggest that because of the reduction in the specific surface area of these UCNPs, the loss of energy from the surface decreases.

**Figure 5:**
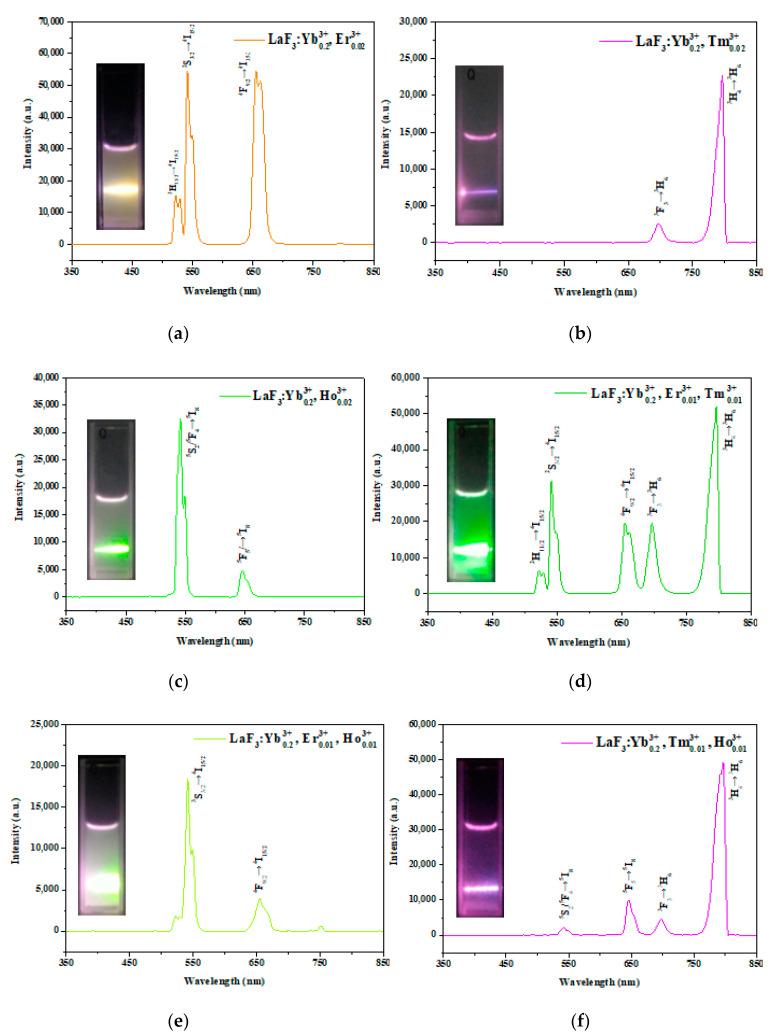

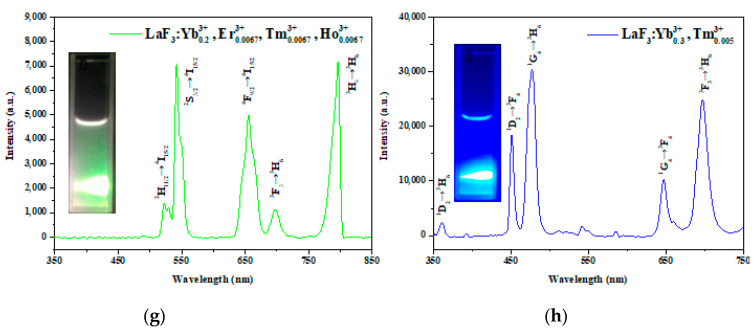
Photoluminescence spectra of LaF_3_:Yb^3+^/Er^3+^/Ho^3+^/Tm^3+^ core nanoparticles with different mole concentrations of Er^3+^, Ho^3+^, and Tm^3+^ under 980 nm near-infrared (NIR) irradiation. The inset in each figure (**a**–**h**) is the corresponding luminescence photograph of the core UCNP in the solution.

As the fluorescence intensities of the UCNPs obtained using 2.5 and 5 mmol NaOH followed by the reaction for 1 h were similar, and the UCNPs achieved using 2.5 mmol NaOH possessed a relatively uniform particle size, defined morphology, and pristine crystal structure, we used these reaction conditions to synthesize the other seven types of UCNPs. For comparison, the reaction parameters and the doping compositions for the eight kinds of UCNPs are listed in Table 1, and their corresponding PL spectra are shown in Figure 5. 

Figure 5a shows the PL spectra of the LaF_3_:Yb^3+^_0.20_, Er^3+^_0.02_ UCNPs with green fluorescence at 523 and 542 nm and red fluorescence at 656 nm, resulting from the ^2^H_11/2_→^4^I_15/2_ (523 nm), ^4^S_3/2_→^4^I_15/2_ (542 nm), and ^4^F_9/2_→^4^I_15/2_ (656 nm) transitions of Er^3+^. The mixed emission appeared orange. The luminescence characteristics of LaF_3_ single-doped with Tm^3+^ were due to ^3^F_3_→^3^H_6_ (700 nm) and ^3^H_4_→^3^H_6_ (800 nm) transitions, as shown in Figure 5b. The mixed red and infrared emissions appeared magenta (inset). In the case of LaF_3_ single-doped with Ho^3+^, the characteristic emission peaks originated from ^5^S_2_/^5^F_4_→^5^I_8_ (542 nm) and ^5^F_5_→^5^I_8_ (645 nm) transitions (Figure 5c), and the mixed emission appeared light green (inset). By comparing Figure 5a,c, we suggest that the dopant Ho^3+^ leads to green fluorescence, while the dopant Er^3+^ results in green and red fluorescence.

Next, the effect of multiple doping (Er^3+^, Ho^3+^, and Tm^3+^) on the PL spectra of the UCNPs was investigated, and the results are shown in Figure 5d–g. The Er^3+^/Tm^3+^, Er^3+^/Ho^3+^, and Tm^3+^/ Ho^3+^ double-doped UCNPs exhibited green, green–pink, and magenta emissions, respectively, resulting from the mixing of their respective emissions. For the Er^3+^/Ho^3+^/Tm^3+^ tri-doped UCNPs, three emission peaks (green, red, and infrared) were observed, and the emitted light was green.

More importantly, to synthesize a kind of UCNPs that can emit UV light and can be applied as a trigger for photoresponsive materials, some preliminary experiments were conducted. As our previous study indicated that UV-emissive LiYF_4_-type UCNPs can be obtained by the single doping of Tm^3+^ [38,44], we optimized the doping concentrations of Yb^3+^ and Tm^3+^ to synthesize LaF_3_ UCNPs with luminescence characteristics in the UV region. As the UC emission is strongly related to the cross-relaxation of the doping ions, we increased the concentration of Yb^3+^ (sensitizer) and decreased the concentration of Tm^3+^ (activator) based on the doping concentrations shown in Figure 5b. 

Consequently, UV-emissive UCNPs were successfully synthesized, as shown in Figure 5h. As shown in Figure 5h, the characteristic peaks of UV emission at 361 nm, blue emission at 451 nm and 477 nm, and red emission at 647 nm and 698 nm originated from ^1^D_2_→^3^H_6_, ^1^D_2_→^3^F_4_, and ^1^G_4_→^3^H_6_, and ^1^G_4_→^3^F_4_ and ^3^F_3_→^3^H_6_ transitions of Tm^3+^, respectively. The inset showed blue emission. Although the intensity of the UV peak was lower than those of the other visible peaks, these UCNPs can still be used to trigger photoresponsive materials for applications, such as controlled drug release [44].

### 3.5. Structural, Morphological, and Optical Properties of the LaF_3_:Yb^3+^_0.20_, Er^3+^_0.02_@ LaF_3_:Yb^3+^_0.20_ Core/Shell UCNPs

**Figure 6:**
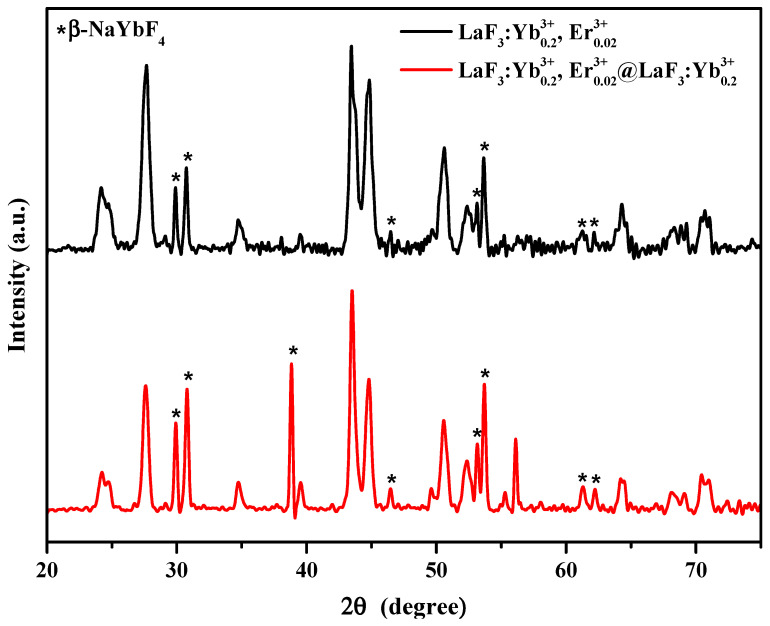
X-ray diffraction patterns for the as-synthesized LaF_3_:Yb^3+^_0.20_, Er^3+^_0.02_ core UCNPs and LaF_3_:Yb^3+^_0.20_, Er^3+^_0.02_@LaF_3_:Yb^3+^_0.20_ core/shell UCNPs (*: β-NaYbF_4_ crystals).

**Figure 7:**
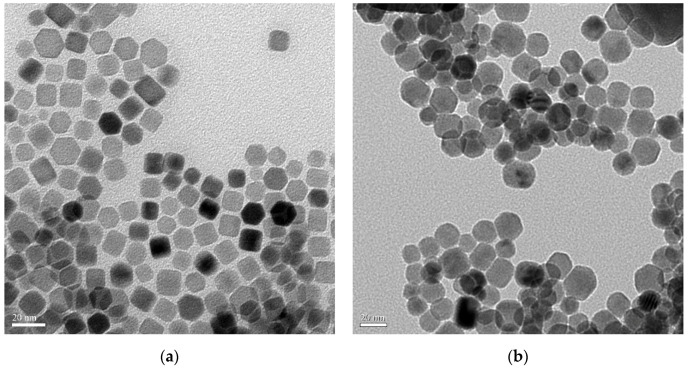
TEM images of (**a**) LaF_3_:Yb^3+^_0.20_, Er^3+^_0.02_ core nanoparticles and (**b**) LaF_3_:Yb^3+^_0.20_, Er^3+^_0.02_@LaF_3_:Yb^3+^_0.20_ core/shell nanoparticles.

**Figure 8:**
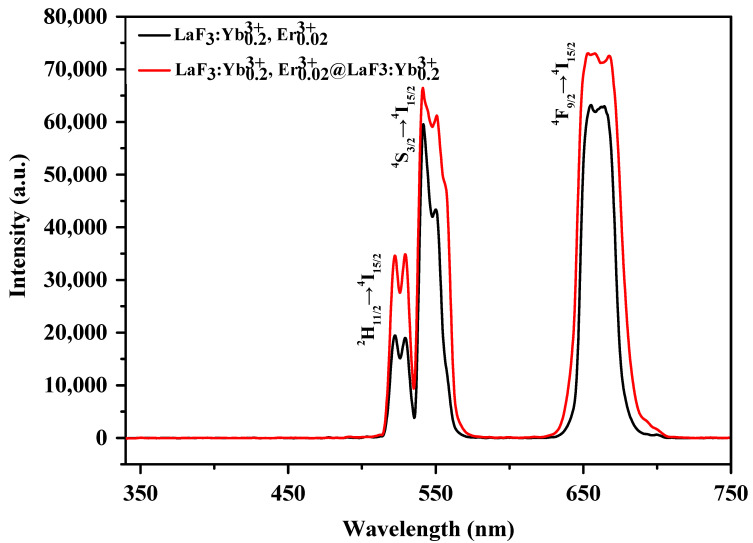
Photoluminescence spectra of LaF_3_:Yb^3+^_0.20_, Er^3+^_0.02_ core UCNPs and LaF_3_:Yb^3+^_0.20_, Er^3+^_0.02_@LaF_3_:Yb^3+^_0.20_ core/shell UCNPs.

As mentioned above, because the core UCNPs prepared using 2.5 mmol NaOH followed by the reaction for 1 h showed a relatively uniform particle size, high fluorescence intensity, defined morphology, and pristine crystal structure, we used these reaction conditions to synthesize LaF_3_:Yb^3+^_0.20_, Er^3+^_0.02_@LaF_3_:Yb^3+^_0.20_ core/shell UCNPs. For comparison, the XRD patterns, TEM images, and PL spectra of the LaF_3_-type core UCNPs and core/shell UCNPs are shown in Figure 6, Figure 7 and Figure 8, respectively.

Figure 6 shows the XRD patterns of the LaF_3_:Yb^3+^_0.20_, Er^3+^_0.02_ core UCNPs and the LaF_3_:Yb^3+^_0.20_, Er^3+^_0.02_@LaF_3_:Yb^3+^_0.20_ core/shell UCNPs. As observed in the figure, the positions of the diffraction peaks before and after the shell coverage are approximately the same, and both UCNPs had hexagonal crystal structures. After coating the core UCNPs with the shell, the FWHMs of the diffraction peaks became narrower, which indicated a larger particle size and crystal growth of the core/shell UCNPs. Although the diffraction peaks of β-NaYbF_4_ were also observed, the formation of β-NaYbF_4_ did not affect the PL spectrum of the LaF_3_ UCNPs, as stated above. The XRD results confirmed the grain growth and successful shell coverage of the core UCNPs.

Figure 7 shows the TEM images of the core UCNPs and core/shell UCNPs at 80,000× magnification. After coating the core UCNPs with the LaF_3_:Yb^3+^ shell, both the side length of the hexagon and the prism height for the core UCNPs significantly increased. Before shell coverage, the mean side length of the hexagon was approximately 9.4 ± 1.0 nm, and the prism height was approximately 11.7 ± 1.0 nm. After shell coverage, the mean side length of the hexagon was approximately 13.5 ± 2.0 nm, and the prism height was ca. 15.6 ± 1.0 nm. The TEM results confirmed that, after shell coverage, the particle size of the UCNPs increased, and core/shell-type UCNPs were successfully synthesized.

Figure 8 shows the PL spectra of the LaF_3_:Yb^3+^_0.20_, Er^3+^_0.02_ core UCNPs and LaF_3_:Yb^3+^_0.20_, Er^3+^_0.02_@LaF_3_:Yb^3+^_0.20_ core/shell UCNPs. As shown in Figure 8, the fluorescence intensities of the LaF_3_:Yb^3+^_0.20_, Er^3+^_0.02_@LaF_3_:Yb^3+^_0.20_ core/shell UCNPs increased in both the green and red regions, after shell coverage; this may be because the reduction in the surface defects of UCNPs led to a decrease in the energy loss during energy transfer.

### 3.6. Detection of Hg^2+^ in Aqueous Media

To detect Hg^2+^ in an aqueous environment, the hydrophobic UCNPs must be converted into hydrophilic colloids via a surface modification approach. As the as-synthesized oleate-capped UCNPs were hydrophobic, they could not be readily dispersed in aqueous solutions. Therefore, the utilization of surface modification techniques was imperative to make these UCNPs water-dispersible for aqueous applications. As PEG exhibits strong hydrophilicity, PEG–imidazole was synthesized and used as a hydrophilic ligand to modify the surfaces of UCNPs via ligand exchange [45,46,47]. 

The Fourier transform infrared (FTIR) spectra of oleate-capped UCNPs and PEG-imidazole capped UCNPs are shown in the Supplementary Materials (Figure S1). The dynamic light scattering (DLS) data of PEG-imidazole capped UCNPs is shown in Figure S2 (Supplementary Materials). After ligand exchange, the water dispersibility of the as-prepared UCNPs was substantially enhanced owing to the formation of hydrogen bonds between the water and oxygen atoms of the PEG chains. The imidazole moiety of PEG–imidazole possesses a pyrrolic nitrogen atom and a pyridinic nitrogen atom. The pyridinic nitrogen atom provided a lone pair of electrons for the coordination of PEG–imidazole to La^3+^ [48]. Consequently, after ligand exchange, the hydrophilic PEG segments were capped to the core/shell UCNPs, allowing the UCNPs to easily disperse in water.

**Figure 9:**
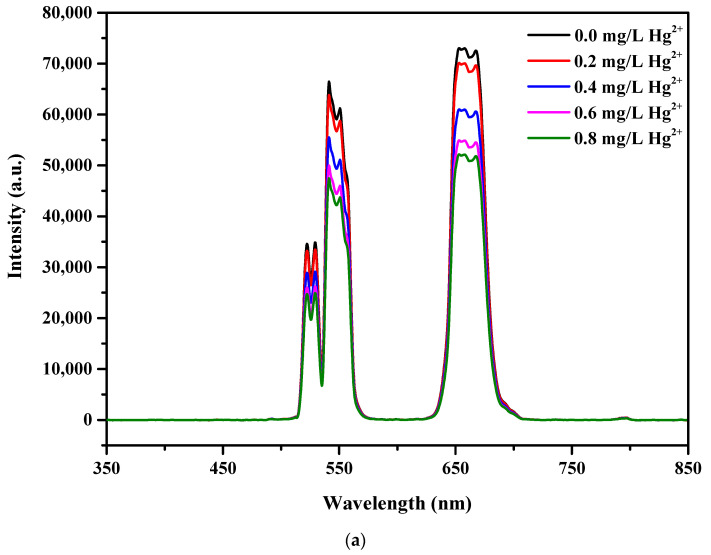

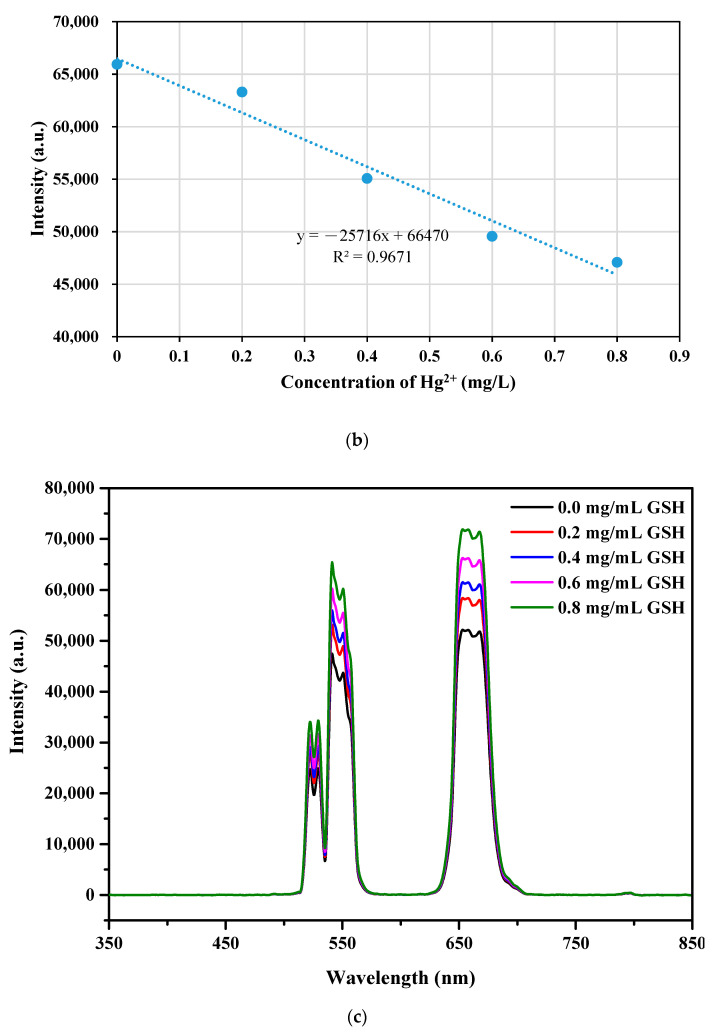

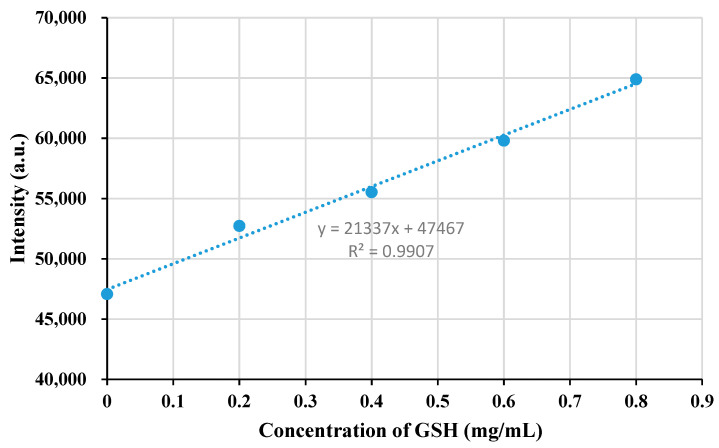
(**a**) Photoluminescence spectra of the LaF_3_:Yb^3+^_0.20_, Er^3+^_0.02_@LaF_3_:Yb^3+^_0.20_ core/shell UCNPs under different Hg^2+^ concentrations and (**b**) linear relationship between the emission intensity and Hg^2+^ concentration. (**c**) Photoluminescence spectra of the UCNPs/Hg^2+^ system under the increasing glutathione (GSH) concentrations and (**d**) linear relationship between the emission intensity and GSH concentration.

Thus, the synthesized LaF_3_:Yb^3+^_0.20_, Er^3+^_0.02_@LaF_3_:Yb^3+^_0.20_ core/shell UCNPs can function as a chemosensor to detect Hg^2+^ in aqueous solutions. As the LaF_3_:Yb^3+^_0.20_, Er^3+^_0.02_@LaF_3_:Yb^3+^_0.20_ core/shell UCNPs exhibited strong green emission, as illustrated in Figure 9, a sensitivity titration was carried out in aqueous media to obtain the standard curve for different Hg^2+^ concentrations in the range from 0 to 0.8 mg/L. After separately adding Hg^2+^ at each concentration to the UCNP solution and irradiating the resulting UCNP solutions with a 980 nm NIR laser, we observed that the intensities of all the emission peaks of UCNPs decreased with an increase in the Hg^2+^ concentration. 

As shown in Figure 9a, upon increasing the concentration of Hg^2+^ for each addition, the intensities of the green and red emissions of UCNPs gradually decreased under 980 nm NIR irradiation, indicating that the present sensing system exhibited high sensitivity for the detection of Hg^2+^. Figure 9b shows a linear relationship between the intensity of UC emissions at approximately 540 nm and the Hg^2+^ concentration. Assuming that the intensity of the green emission band can be reliably detected to the amount as low as 0.1 mg/L based on the variation of the PL intensity and from the standard curve, it might be reasonable that the accepting detection level of Hg^2+^ ions is approximately 0.1 mg/L or 0.5 µM.

To employ the developed fluorescent sensor for the quantitative detection of GSH, further investigation was performed to analyze the fluorescence response of the UCNPs/Hg^2+^ system with the addition of GSH. We found that, after adding a certain amount of GSH to the UCNPs/Hg^2+^ system, the fluorescence intensity immediately recovered (Figure 9c). When the concentration of GSH was increased from 0 to 0.8 mg/mL, the fluorescence intensities of the UCNPs increased. Figure 9d shows a linear relationship between the fluorescence intensity of the UCNPs measured at approximately 540 nm and GSH concentration in the range from 0 to 0.8 mg/mL.

The results of Hg^2+^ sensing and fluorescence quenching can be explained according to the following phenomenon: when Hg^2+^ was present in close proximity to the UCNPs, UCNP–Hg^2+^ complexes were formed. The fluorescence intensities of the UCNPs decreased due to the formation of the UCNP–Hg^2+^ coordination complexes, which caused the static quenching of green emission via electron transfer from the LaF_3_:Yb^3+^_0.20_, Er^3+^_0.02_@LaF_3_:Yb^3+^_0.20_ UCNPs to Hg^2+^ [49,50,51,52]. The static quenching mechanism can result in linear Stern-Volmer plots as shown in Figure S3 of the Supplementary Materials. 

Except for the electron transfer, the ion-bonding may lead to PL quenching; however, the former effect may play a more important role [50]. Upon increasing the loading of Hg^2+^, the fluorescence intensities of the UCNPs decreased, whereas the fluorescence quenching increased. However, the fluorescence intensity could be recovered after adding a certain amount of GSH to the UCNPs/Hg^2+^ solution. As GHS and Hg^2+^ formed a stable Hg–S bond [53,54], the UCNP–Hg^2+^ coordination complexes were removed, leading to the recovery of the fluorescence intensity.

## 4. Conclusions

In this study, various hexagonal-phase LaF_3_:Yb^3+^/Er^3+^/Ho^3+^/Tm^3+^ UCNPs were prepared by single-doping, double-doping, and triple-doping LaF_3_:Yb^3+^ using UC lanthanide ions via the thermal decomposition approach. In particular, LaF_3_:Yb^3+^_0.20_, Er^3+^_0.02_@LaF_3_:Yb^3+^_0.20_ core/shell UCNPs with a hexagonal prism structure, a uniform size, and bright UC luminescence were obtained. The effects of the amount of NaOH and reaction time on the crystal structure and particle size of these UCNPs were studied in detail. By adjusting the conditions for the synthesis of Yb/Er co-doped LaF_3_ nanocrystals, pure hexagonal-phase UCNPs were obtained by adding 5.0 mmol NaOH to the reaction mixture followed by reacting for 30 min. 

In addition, the fluorescence intensities of the UCNPs were up to 17 times that of the control sample when 2.5 and 5.0 mmol NaOH and a reaction time of 1 h were employed. By modifying the composition and concentration of the dopants, the UC emissions obtained under 980 nm NIR laser irradiation could be precisely adjusted. By tuning the doping ratio of Yb/Tm, UV-emissive LaF_3_:Yb^3+^_0.30_, Tm^3+^_0.005_ UCNPs were successfully synthesized. The hydrophobic LaF_3_:Yb^3+^_0.20_, Er^3+^_0.02_@LaF_3_:Yb^3+^_0.20_ core/shell UCNPs capped using oleic acid were surface-modified with PEG–imidazole via ligand exchange and converted into water-dispersible UCNPs. The present system was effectively applied in the detection of Hg^2+^ under NIR irradiation.

## Figures and Tables

**Table 1:** The concentration of doping ions in LaF_3_:Yb^3+^/Er^3+^/Ho^3+^/Tm^3+^ upconversion nanoparticles.

Nanoparticle Samples		Ion Concentration (mmol)			
		Yb^3+^	Er^3+^	Ho^3+^	Tm^3+^
No. 1	LaF3:${\mathrm{Yb}}_{0.20}^{3+}$/${\mathrm{Er}}_{0.02}^{3+}$	0.20	0.02	0	0
No. 2	LaF3:${\mathrm{Yb}}_{0.20}^{3+}$/${\mathrm{Tm}}_{0.02}^{3+}$	0.20	0	0	0.02
No. 3	LaF3:${\mathrm{Yb}}_{0.20}^{3+}$/${\mathrm{Ho}}_{0.02}^{3+}$	0.20	0	0.02	0
No. 4	LaF3:${\mathrm{Yb}}_{0.20}^{3+}$/${\mathrm{Er}}_{0.01}^{3+}$/${\mathrm{Tm}}_{0.01}^{3+}$	0.20	0.01	0	0.01
No. 5	LaF3:${\mathrm{Yb}}_{0.20}^{3+}$/${\mathrm{Er}}_{0.01}^{3+}$/${\mathrm{Ho}}_{0.01}^{3+}$	0.20	0.01	0.01	0
No. 6	LaF3:${\mathrm{Yb}}_{0.20}^{3+}$/${\mathrm{Ho}}_{0.01}^{3+}$/${\mathrm{Tm}}_{0.01}^{3+}$	0.20	0	0.01	0.01
No. 7	LaF3:${\mathrm{Yb}}_{0.20}^{3+}$/${\mathrm{Er}}_{0.0067}^{3+}$/${\mathrm{Ho}}_{0.0067}^{3+}$/${\mathrm{Tm}}_{0.0067}^{3+}$	0.20	0.0067	0.0067	0.0067
No. 8	LaF3:${\mathrm{Yb}}_{0.30}^{3+}$/${\mathrm{Tm}}_{0.005}^{3+}$	0.30	0	0	0.005

## Supplementary Materials

Supplementary materials can be found at https://www.mdpi.com/2079-4991/10/12/2477/s1. Figure S1: Fourier transform infrared spectra of oleate-capped UCNPs and polyethylene glycol (PEG)-imidazole capped UCNPs (after ligand exchange). Figure S2: Dynamic light scattering (DLS) analysis of the LaF_3_:Yb^3+^_0.20_, Er^3+^_0.02_@LaF_3_:Yb^3+^_0.20_ core/shell UCNP. Figure S3. Stern-Volmer plots of two main emission bands for Hg^2+^ induced quenching of the LaF_3_:Yb^3+^_0.20_, Er^3+^_0.02_@LaF_3_:Yb^3+^_0.20_ UCNPs.
