# Supplementary material for: Synthesis, Optical Properties, and Sensing Applications of LaF3:Yb3+/Er3+/Ho3+/Tm3+ Upconversion Nanoparticles
Source: Nanomaterials (Basel). 2020 Dec 10;10(12):2477. doi: 10.3390/nano10122477 (PMC7774313; doi:10.3390/nano10122477)
Supplement: Supplementary file 1 [file nanomaterials-10-02477-s001.pdf]

Supplementary Materials

Synthesis, Optical Properties, and Sensing Applications of $LaF_3:Yb^{3+}/Er^{3+}/Ho^{3+}/Tm^{3+}$ Upconversion Nanoparticles

Hsiu-Wen Chien [1,*], Chien-Hao Huang [2], Chien-Hsin Yang [2] and Tzong-Liu Wang [2,*]

[1] Department of Chemical and Materials Engineering, National Kaohsiung University of Science and Technology, Kaohsiung 807, Taiwan

[2] Department of Chemical and Materials Engineering, National University of Kaohsiung, Kaohsiung 811, Taiwan; yellow1000mm@gmail.com (C.-H.H.); yangch@nuk.edu.tw (C.-H.Y.)

* Correspondence: hsiu-wen.chien@nkust.edu.tw (H.-W.C.); tlwang@nuk.edu.tw (T.-L.W.); Tel.: +886-7-381-4526 (ext. 15124) (H.-W.C.); +886-7-591-9278 (T.-L.W.); Fax: +886-7-383-0674 (H.-W.C.); +886-7-5919277 (T.-L.W.)

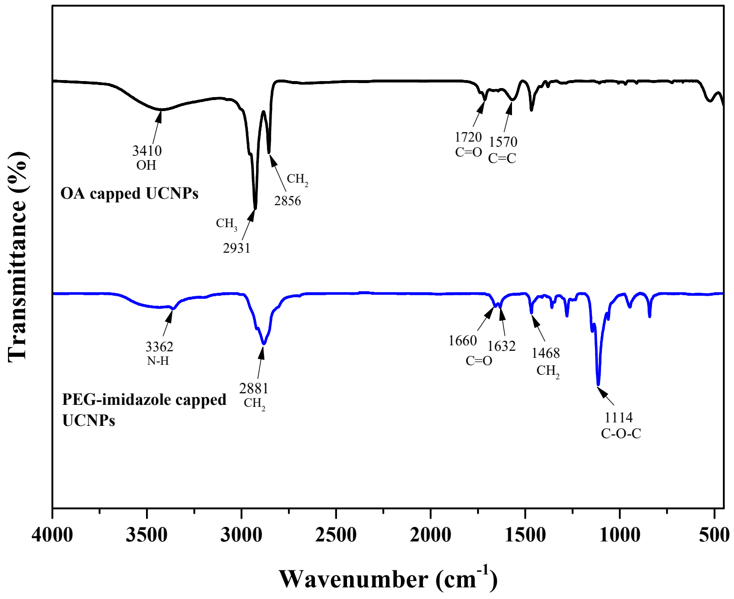

Figure S1. Fourier transform infrared spectra of oleate-capped UCNPs and PEG-imidazole capped UCNPs (after ligand exchange).

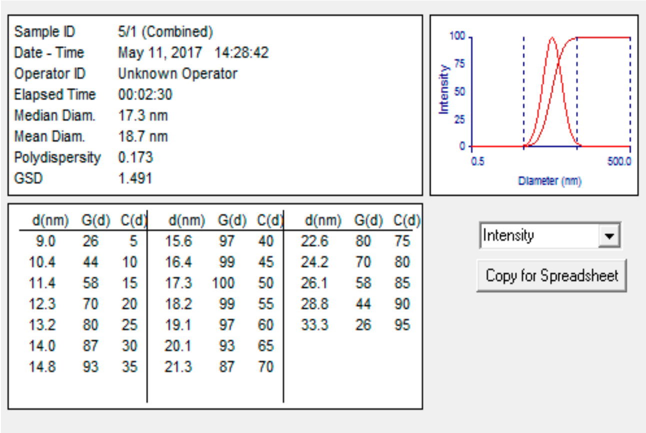

Figure S2. Dynamic light scattering (DLS) analysis of the LaF_3:$Yb^{3+}_{0.20}$,$Er^{3+}_{0.02}$@LaF_3:$Yb^{3+}_{0.20}$ core/shell UCNP after ligand exchange.

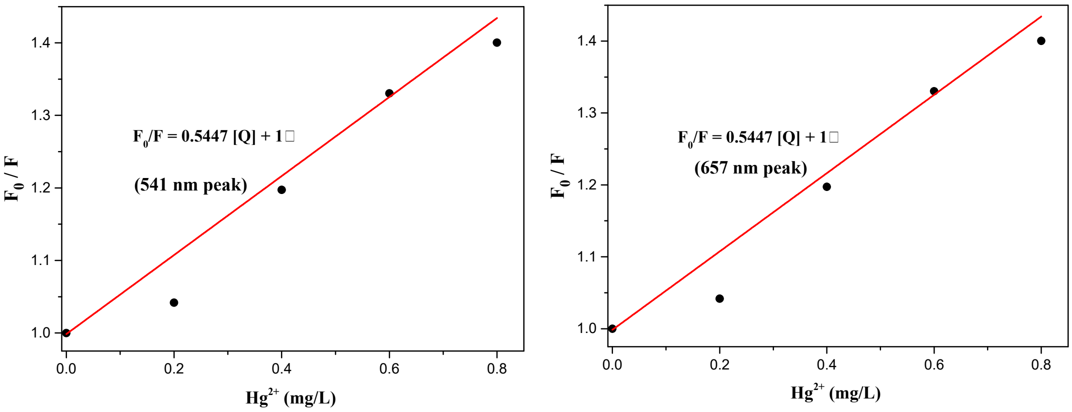

Figure S3. Stern-Volmer plots of two main emission bands for Hg^{2+} induced quenching of the LaF_3:$Yb^{3+}_{0.20}$,$Er^{3+}_{0.02}$@LaF_3:$Yb^{3+}_{0.20}$ UCNPs.
